# Ultrasonication of Thawed Huyou Juice: Effects on Cloud Stability, Physicochemical Properties and Bioactive Compounds

**DOI:** 10.3390/foods10081695

**Published:** 2021-07-22

**Authors:** Xinyue Zhou, Wenjun Wang, Xiaobin Ma, Enbo Xu, Donghong Liu

**Affiliations:** 1 Zhejiang R&D Center for Food Technology and Equipment, Zhejiang Key Laboratory for Agro-Food Processing, College of Biosystems Engineering and Food Science, Zhejiang University, Hangzhou 310058, China; xinyuez@zju.edu.cn (X.Z.); xbma@zju.edu.cn (X.M.); enboxu@zju.edu.cn (E.X.); 2 Ningbo Research Institute, Zhejiang University, Ningbo 315100, China; 3 Fuli Institute of Food Science, Zhejiang University, Hangzhou 310058, China

**Keywords:** thawed Huyou juice, ultrasonication, cloud stability, properties, nutritional quality

## Abstract

In order to remove the flocculent precipitation in Huyou juice after frozen storage and thawing process, the thawed juice was ultrasonically treated with different power (45–360 W) and time (10–60 min) in ice bath (~0 °C), and its sedimentation behavior during storage was observed. After optimization, the cloud stability of juice could be improved by ultrasonic treatment with ultrasonic power of 360 W or more for at least 30 min, which could be stable during 7 days of storage at 4 °C. Under this optimal condition (360 W, 30 min), the effects of ultrasound on the physicochemical properties and bioactive compounds of thawed Huyou juice during storage were investigated. The results showed that with smaller particle size and lower polymer dispersity index, ultrasonic treatment did not significantly change the color, soluble solids, titratable acidity, and bioactive compounds including flavonoids and other phenolics. In addition, all properties of samples were at the same level during storage. Thus, ultrasound was applicable since it can improve the cloud stability of Huyou juice with minimal impact on its physicochemical properties and nutritional quality compared to the untreated one.

## 1. Introduction

Huyou (*Citrus aurantium* ‘*Changshanhuyou*’), originating in Quzhou, Zhejiang province, is one of the main citrus species in China [1]. It is a hybrid between sour pomelo and orange lime and rich in nutrients like nootkatone, scopone, nobiletin, naringin, hesperidin, neohesperidin, limonin and nomilin [2,3]. These nutrients have many biological activities, such as anti-inflammatory [3], anti-oxidant [2,4], and anti-bacterial activities [5]. Currently, Huyou has been made into a variety of products, such as essential oils [5], traditional Chinese medicine Qu Zhi Ke [6], and juice [7].

Cloudy juice is fast-growing, among which Huyou juice (fresh juice, not from concentrate), rich in naringin, neohesperidin and total flavanone glycosides [7], is a typical example. Besides flavor and color, higher cloud stability of juice without sediment is preferred more by consumers [8]. In addition, the cloud of juice also makes contributions to color, flavor and nutrition [9]. It was found that the cloud in citrus juice was chromo plastids, pulp or cell wall fragments, spherical oil droplets, and needle-like crystals, containing considerable protein and carbohydrate [8]. The existence of pectin, pectin methyl esterase, turbidity agent like protein, particle size of suspension, and potential would all affect the cloud stability of fruit juice [9,10,11]. So far, the methods for improving the cloud stability of juice mainly included high pressure homogenization [12], continuous high pressure carbon dioxide processing [13] and thermal treatments [14].

Freezing is a conventional fresh-keeping method for maintaining quality of fresh juice. However, in the factory’s previous production practice, after being filtered roughly and stored in a bucket with a volume of 30 L at –18 °C, the Huyou juice was found to flocculate and precipitate while being thawed at room temperature, resulting in the loss of commercial value. A similar phenomenon has also been reported that a decrease in color intensity and deterioration in appearance tended to occur for juice in the process of freezing and thawing. It was also found that when the freezing rate was slow, phenomena including sediment of insoluble particles disintegration of plastids, and color change would be observed [15].

Ultrasonication is nonthermal processing technology, which releases energy by chemical and mechanical effects through the collapsing of cavitation bubbles, generated by a series of compression and rarefaction of ultrasound wave [16]. In the case of juice, its use has been verified in several studies. For example, power ultrasound treatment had a great potential to increase the amount of bioactive compounds and antioxidants capacity of kiwifruit juice, strawberry juice and cape gooseberry juice [17,18,19]; ultrasound in combination with other preservation techniques such as heating at mild temperature, processing under moderate pressure, and using antimicrobials, could inactivate microorganisms in juice with minimal impact on physicochemical and nutritional properties [20,21]; thermosonication was reported to not only improve the juice homogeneity with little color change but also increase the total soluble solids and titratable acidity [22].

The objectives of this study were (1) to use ultrasonic technology to improve the homogeneity and cloud stability of Huyou juice during storage; (2) to optimize the power and time of sonication based on the lowest sedimentation behavior of the juice; (3) to study the effects of ultrasonication on the physicochemical properties and nutritional quality of Huyou juice by comparing the color and the contents of soluble solids, titratable acidity and bioactive compounds between the treated and untreated juice after 0 and 7 days of storage at 4 °C.

## 2. Materials and Methods

### 2.1. Materials

Frozen and fresh Huyou juice (not from concentrate) were kindly provided by a company in Quzhou, China. Among them, frozen juice was stored in a bucket with a volume of 30 L at −18 °C, and fresh juice was packed in plastic bags with volumes of 1 L at 4 °C.

### 2.2. Ultrasonic Treatment

A series of juice was thawed at room temperature and stirred evenly, and then was sonicated by a probe sonicator (JY92-IIDN, Scientz, Ningbo, Zhejiang, China) coupled with a probe with a diameter of 6 mm under different power (45–360 W) for different time (10–60 min). For each ultrasound treated group, 200 mL of juice in the beaker with the diameter of 75 mm and volume of 250 mL was treated with ultrasound at 22 kHz. The temperature of the samples was maintained by an ice bath and they were stored in four 50 mL centrifuge tubes at 4 °C during the storage.

### 2.3. Observation and Measurement of Sedimentation Behavior

The sedimentation behavior of Huyou juice was recorded with photos under a standard light source (TL84, T60(5), Tilo, Shenzhen, China) and measured by the natural sedimentation percentage according to Xingqian [23], which was expressed as the ratio of the volume of the supernatant after 3 or 6 days of storage to the total volume of the juice.

### 2.4. Color Analysis

The juice color was measured by a colorimeter (Colorflex-EZ, HunterLab, Reston, VA, USA) with CIE LAB system by reporting lightness (L*), redness (a*), and yellowness (b*). A black plate and a white plate were used as calibration references. All the measurements were performed after stirring juice evenly.

### 2.5. Total Soluble Solids (TSS) and Titratable Acidity (TA)

TSS and TA were measured by a portable sugar and acid meter (PAL-BX|ACID F5, ATAGO, Tokyo, Japan). TA was determined using 50-fold dilution of supernatant of juice. 

### 2.6. Antioxidant Activity

#### 2.6.1. 1,1-Diphenyl-2-picrylhydrazyl (DPPH) Radical Scavenging Activity

DPPH radical scavenging activity was determined based on the method of Gorinstein et al. [24] with slight modifications. In total, 2.8 mL of a 0.04 g/L DPPH ethanol solution was added to a 0.2 mL aliquot of 3-fold dilution of juice supernatant (or ascorbic acid at 0–0.15 mg/mL). The absorbance was detected at 517 nm after incubation at 25 °C for 30 min. Percentage of the decrease in absorbance of the samples compared with the blank was used to calculate the radical-scavenging activity. The result was expressed as ascorbic acid equivalents (AAE).

#### 2.6.2. The Ferric Reducing Ability of Plasma (FRAP)

Antioxidant activity of Huyou juice was also assessed with FRAP assay [25,26]. Firstly, FRAP reagent was made by mixing 20 mmol/L FeCl_3_, 10 mmol/L 4,6-tripryridyl-s-triazine (TPTZ, dissolved in 40 mmol/L HCl), and 100 mmol/L acetate buffer (pH 3.6, 10:1:1, *v/v/v*). A total of 4.9 mL of FRAP reagent was added to 0.1 mL of 2.33-fold dilution of juice supernatant. After incubating in the dark for 30 min, the absorbance of the samples was detected at 593 nm. The result was reported as trolox equivalent antioxidant capacity (TE) according to the calibration curves prepared by trolox solution.

### 2.7. Phenolic Content

#### 2.7.1. Total Phenolics Content

The total phenolics content was measured according to Folin–Ciocalteu (FC) colorimetric assay with slight modifications [27,28]. Folin–Ciocalteu phenol reagent was equally mixed with 3-fold dilution of juice supernatant (or gallic acid standard at 0–10 mg/mL). After 2 mL of the mixtures were vortexed, they were then rested for 5 min at 20 °C. Then, 5 mL of Na_2_CO_3_ solution (5%, *w/v*) and 18 mL of distilled water were added. After incubation in the dark for 60 min, the absorbance of the samples at 765 nm was read. The result was expressed as gallic acid equivalents (GAE).

#### 2.7.2. Total Flavonoids Content

The total flavonoids content was measured according to Huang et al. [29] with some modifications. Briefly, 5.0 mL of 6-fold dilution of juice supernatant (or rutin standard at 0–25 μg/mL) was reacted with 0.3 mL of 5% NaNO_2_ (*w/v*) for 5 min. Then, 0.3 mL of 10% Al(NO_3_)_3_ (*w/v*) was added to the mixture. After 6 min, 4 mL of 1 mol/L NaOH was added for another incubation of 15 min. After measuring the absorbance of the samples at 510 nm, the result was reported in rutin equivalent (RE).

#### 2.7.3. Flavanone Composition

The flavanone composition was measured according to Huang et al. [29] with some modifications. Briefly, juice was diluted 2.33 times with methanol and flavonoid standards (eriocitrin, neoeriocitrin, narirutin, naringin, neohesperidin from Sigma-Aldrich (St. Louis, MO, USA)) were dissolved in methanol and sufficiently mixed. After filtration through a PTFE membrane with φ = 13 mm and pore size of 0.45 mm, 10 μL of samples (or standard solutions) were injected into the HPLC system (Waters e2695, Waters, Milford, MA, USA) coupled with a UV/vis detector (Waters 2489, Waters, Milford, MA, USA). The XDB-C18 column (250 × 4.6 mm, Agilent, Santa Clara, CA, USA) was kept at 25 °C with a flow rate of 0.7 mL/min. Solution A (0.1% formic acid) and solution B (methanol) were used as the mobile phase with the gradient elution as follows: 0–20 min, 63–50% A; 20–25 min, 50–20% A; 25–30 min, 20–0% A; 30–35 min, 0% A; 35–40 min, 0–63% A; 40–42 min, 63% A. The detection was performed at 283 nm. The flavonoids were identified and quantified according to the standard curves (Figure S1).

### 2.8. Particle Size Distribution and Zeta-Potential

The particle size (mean diameter), polymer dispersity index (PDI), and zeta-potential were analyzed by Zetasizer (Nano ZS90, Malvern, UK). The samples were dispersed in distilled water and equilibrated for 60 s at 25 °C before being measured. The water refraction index was 1.33, the viscosity was 0.8872 cP, the dielectric constant was 78.5, and the particle refraction index was 1.5 [30]. 

### 2.9. Optical Microscopic Observation

The juice samples were observed by an optical microscope (UPH203i, UOP, Chongqing, China) equipped with a camera (JFMV-M1200C, JIFEI, Nanjing, China). Approximately 20 μL of juice sample were mounted onto the glass slide.

### 2.10. Experimental Design and Statistical Analysis

**Figure 1:**
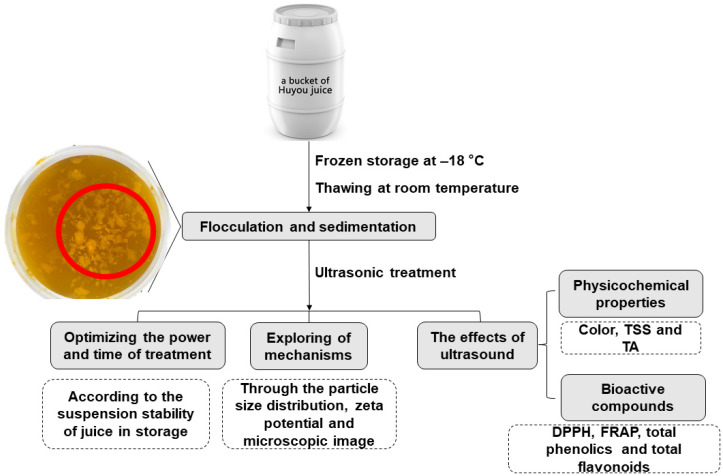
Diagram showing the practical problem and experimental design.

The single-factor design was applied for investigating the effect of ultrasonic power and treatment time on the cloud stability of juice during storage. Firstly, the effect of ultrasonic power (45, 90, 180, 270, and 360 W) for 10 min was investigated. Secondly, since the juice treated with ultrasonic power of 360 W could alleviate the sedimentation phenomenon most but may need more treatment time, effect of treatment time (10, 20, 30, 40, 50, and 60 min) was further investigated with the ultrasonic power of 360 W. Based on the single-factor experiment, the optimal condition was used for exploring the mechanisms for this phenomenon and comparing physicochemical properties and nutritional quality with the untreated group (Figure 1). 

Data were treated through one-way analysis of variance at the significance level of 0.05 by SPSS 26 based on the Duncan test.

## 3. Results

### 3.1. Cloud Stability

**Figure 2:**
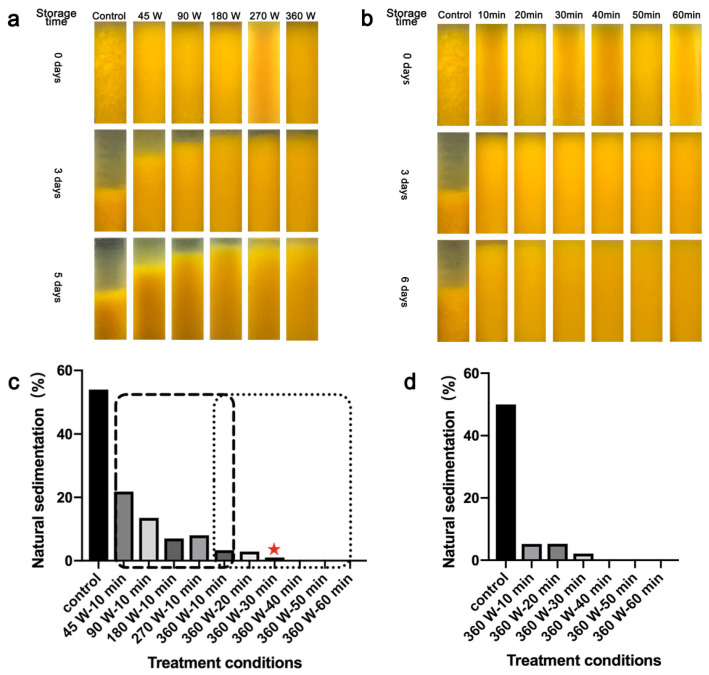
The sedimentation behavior of Huyou juice during storage after different treatments. (**a**) Appearance of thawed Huyou juice during storage after ultrasonic treatments for 10 min with different ultrasonic power. Thawed Huyou juice without ultrasonic treatment was used as the control; (**b**) appearance of thawed Huyou juice during storage after ultrasonic treatments for different time with ultrasonic power of 360 W. Thawed Huyou juice without ultrasonic treatment was used as the control; (**c**) the natural sedimentation percentage of thawed Huyou juice after 3 days of storage after ultrasonic treatments with different power and different time. Thawed Huyou juice without ultrasonic treatment was used as the control. Red star indicated the optimal condition (360 W, 30 min) based on the lowest sedimentation behavior; (**d**) the natural sedimentation percentage of thawed Huyou juice after 6 days of storage after ultrasonic treatments with ultrasonic power of 360 W and different time. Thawed Huyou juice without ultrasonic treatment was used as the control.

The sedimentation behavior of juice during storage after thawing and ultrasonic treatment is shown in Figure 2, reflecting the cloud stability of juice. All thawed juice treated with ultrasound for 10 min showed homogenous appearance at day 0, which was similar to the fresh juice (Figure S2). After 3 days of storage, the sedimentation behavior of thawed juice appeared but could be alleviated with the increase of ultrasonic power, since the sedimentation percentage dropped from 19.0% to 3.8% (Figure 2a,c). Improvements in cloud stability with the increase of ultrasonic power were also found in apple juice [31] and orange juice [32], which may be because of the reduction in the particle size [31], the degradation of linear pectin molecule [32,33] and the inactivation of enzyme [32,34] by ultrasonication. However, the juice treated with ultrasonic power of 360 W showed visible phenomenon of sedimentation with a percentage of 3.8%, which could be due to short treatment time (10 min). At day 5, the separation of insoluble particles from the juice serum was not significantly different from day 3 (Figure 2a).

By using the ultrasonic power of 360 W for 30 min and more, the sedimentation percentage reduced to 1.0% and even 0% (Figure 2c). In other words, the cloud stability of the juice improved with the increasing treatment time (Figure 2b), which was in accordance with previous studies [31,32]. In addition, no obvious sedimentation behavior was found in the treated group for more than 30 min after 6 days of storage at 4 °C (Figure 2b), with the sedimentation percentage of 2.1% (30 min) and even 0% (≥40 min) (Figure 2d).

Generally, the ultrasonic treatment with the power of 360 W for 30 min can make Huyou juice achieve high cloud stability. Therefore, considering the effect of ultrasound and the concept of energy saving, this condition was chosen for the subsequent experiments to explore the mechanisms for this phenomenon and compare physicochemical properties and nutritional quality of ultrasound treated juice with the untreated one.

### 3.2. Particle Size and Zeta Potential

#### 3.2.1. Particle Size Distribution and Microscopic Images

The particle size and polymer dispersity index (PDI) are shown in Table 1. The average particle size of FJ (fresh juice) and US0 (thawed and ultrasonicated juice) were close (*p* > 0.05) and around 2330 nm. However, both of them were significantly smaller than that of TS0 (thawed juice) (*p* < 0.05), which was about 3483 nm. Moreover, the PDI of TS0 was significantly higher than that of FJ (*p* < 0.05), which could be reduced again in US0 (*p* < 0.05). PDI represents the homogeneity of the samples, that is, a smaller PDI value indicates more homogenous juice. It can be inferred that the particle size of the juice will increase and become a wider particle size distribution during the process of freezing and thawing, while ultrasonic treatment can disaggregate larger particles into smaller ones. Moreover, although the size of the FJ and US0 were not very different, the PDI of the US0 was lower than that of FJ significantly, indicating that ultrasonication gave the juice the potential to be even more uniform than the fresh one. The change in particle size could result in the difference in sedimentation behavior of juice, since previous studies found that the smaller the particle size and the more uniform distribution the juice had, the more stable it will be [8,11,35]. However, since all of the samples did not approach the sizes reported to be stable in juice (500–2000 nm) [8], precipitation would happen during a long period of storage.

**Figure 3:**
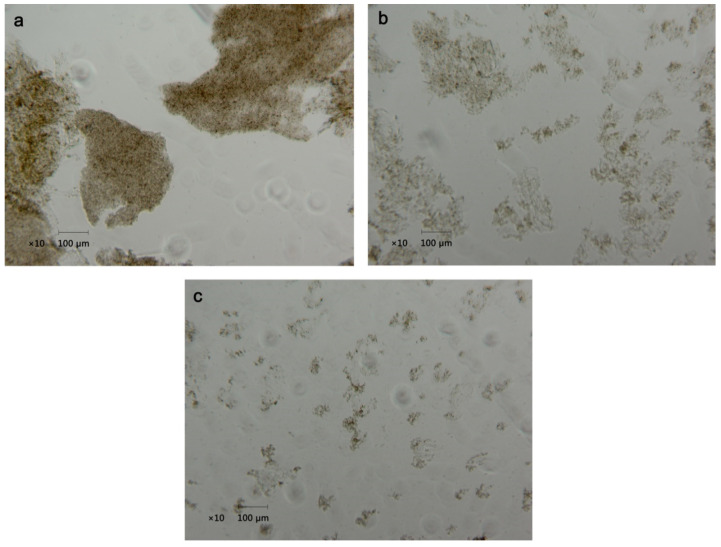
Microscopic images of Huyou juice with different treatments. (**a**) Thawed Huyou juice without ultrasonic treatment; (**b**) thawed Huyou juice which was treated with ultrasonic power of 360 W for 30 min; (**c**) fresh Huyou juice.

**Figure 4:**
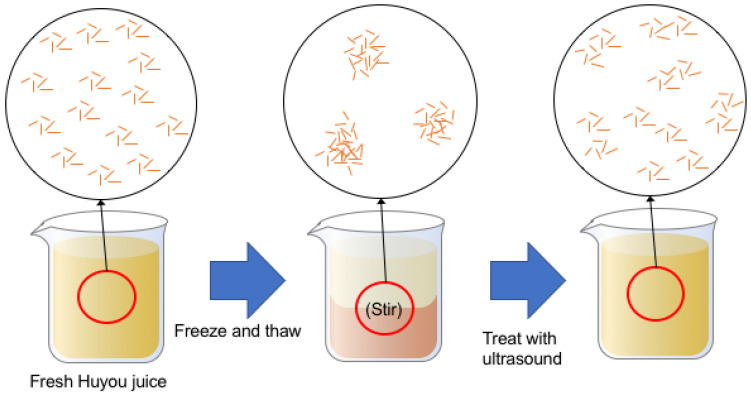
Diagram illustrating the changes of micro particles in Huyou juice after freeze–thaw and ultrasonic treatment.

The morphology of the juice under an optical microscope is shown in Figure 3. It can be seen from the images that the insoluble solids in fresh juice presented a small volume, light color, and evenly dispersed state (Figure 3c), indicating a relatively homogeneous dispersion. However, after freezing and thawing, the solids turned into darker and bigger clumps with larger and clearer gaps (Figure 3a). The flocculation after freezing and thawing has also been observed in orange juice, coconut milk and nanofluid emulsion [15,36,37], which may be because the ice crystals compressed and agglomerated the insoluble particles but thawing could not disperse the aggregates [15]. Moreover, after ultrasonic treatment, the clumps became lighter ones with decreased volume and more even dispersion, although it was still more clustered compared with fresh juice (Figure 3b). A diagram illustrating the changes of micro particles in Huyou juice after freeze–thaw and ultrasonic treatment is shown in Figure 4. This homogenization effect was widely verified and was ascribed to the cavitation and shear stress generated by ultrasound propagation [38,39,40].

#### 3.2.2. Zeta Potential

Zeta potential characterizes the charge of the particle surfaces, affecting the precipitation of juice [41,42]. Research has shown that the stronger the surface charge, the greater the repulsion between particles, and the lower the tendency for aggregation between particles [10,11,42,43]. However, the zeta potential of Huyou juice under the three treatments varied from –7.28 to –6.19 with little difference (*p* > 0.05) (Table 1). Therefore, the change of the juice stability could not be attributed to the difference in particle charge and electrostatic force. Moreover, from the viewpoint of zeta potential, all samples were easy to agglomerate, since the absolute value of zeta potential should be at least 25 mV to provide repulsive force strong enough to overcome the attraction between the particles [10].

### 3.3. Physicochemical Properties

The color of treated juice during storage is shown in Table 1. There was almost no significant difference in all color parameters between thawed juice with and without ultrasonic treatment, while only a* showed lower value after ultrasonication (*p* < 0.05), which could be due to the homogenization effect of ultrasound. Similarly, Gao et al. [39] observed that ultrasonic treatment increased L* of tomato juice and decreased a*, but had little effect on b*. It was suggested that the smaller particles obtained by ultrasonic shearing enhanced the light reflection and thus increased the gloss of the juice [39,44]. Merin et al. believed that the flocculation after the frozen storage was the reason to cause the color change measured by the instrument [15]. Accordingly, in this study, the color change recorded by the colorimeter may be due to the more uniform juice and the smaller insoluble particles, which enhanced the light reflection. Besides, there was no significant difference in all of these values after 7 days of storage (*p* > 0.05), which meant ultrasonic treatment would not influence the color of juice during storage. The natural pigments in Huyou are mainly composed of flavonoids [45], which was stable during storage and is discussed in Section 3.4. Similar results were found in other studies that ultrasound did not reduce the color stability as well. Yildiz et al. [46] observed that the a* and b* of fresh and ultrasonicated strawberry juice were both stable during storage, and ultrasonic treatment could slow down the decrease in L* of strawberry juice. Zia et al. [47] observed that after ultrasonic treatment, the color change of the sugarcane juice was slightly smaller than that of the control. Overall, the effect of ultrasound will not destroy the pigments in the juice, nor will it affect the storage stability.

As shown in Table 2, regarding TA and TSS, the decrease after ultrasonic treatment was rather slight, and there was no significant difference during storage (*p* > 0.05) for both treated and untreated groups. The results were in agreement with research focusing on other ultrasound treated strawberry juice [48], carrot juice [49], orange juice [32,50] and apple juice [51], since soluble solids and acids in juice would stay in juice after sonication and during storage [19,52].

### 3.4. Bioactive Compounds

The percentage of DPPH radical scavenging and FRAP are shown in Table 3. Both antioxidant activities of the juice were not impacted and had a high stability during storage regardless of whether the juice was treated with ultrasound or not (*p* > 0.05). Similar results were also observed in previous studies [53]. It has been confirmed previously that antioxidant activity and phenolic content was positively highly correlated as well [54,55,56]. Additionally, flavonoids are one of the most important groups of phenolic compounds. As shown in Table 3 and Table 4, the total phenolics content, the total flavonoids content and flavanone composition of the juice did not change significantly after ultrasonic treatment or storage (*p* > 0.05). It could be concluded that most active substances had good stability during storage, which would not be influenced by ultrasonic treatment. The similar results could be seen in previous studies as well that thawing and mild ultrasound treatment had little impact on most bioactive compounds in juice [22,53,57].

## 4. Conclusions

It could be concluded that for 200 mL of thawed Huyou juice, ultrasonic treatment with ultrasonic power of 360 W for 30 min could make it come back to homogenous and stable cloudy state during 7 days of storage at 4 °C. The higher average particle sizes and larger PDI of untreated thawed juice with dark aggregates under an optical microscope indicated agglomeration of soluble and insoluble solids induced by freezing and thawing. However, ultrasonication disaggregated large clumps, thus causing the difference in the sedimentation behavior of the juice during storage. Besides, almost no significant changes in physicochemical properties including color, TSS, TA and bioactive compounds such as the contents of phenolics, flavonoids and the antioxidant activity were found after ultrasonic treatment. Above all, ultrasound could improve the appearance and stability of thawed juice meanwhile not affect physicochemical properties and nutritional quality of the juice, showing beneficial potential for its application. Future study could focus on investigating the effect of ultrasonic processing on thawing frozen Huyou juice in order to obtain short thawing time and good cloud stability of juice during one treatment.

## Figures and Tables

**Table 1:** The effect of ultrasonic treatment and storage on of size, PDI and zeta potential of thawed Huyou juice.

Samples	Size (nm)	PDI	Zeta Potential (mV)
FJ	2331 ± 316 ^a^	0.337 ± 0.119 ^b^	–6.19 ± 0.70 ^a^
TS0	3483 ± 896 ^b^	1.000 ± 0.001 ^c^	–7.28 ± 1.33 ^a^
US0	2333 ± 469 ^a^	0.168 ± 0.119 ^a^	–6.95 ± 1.56 ^a^

Mean ± SD indicated three replicates. Superscripts of different letters indicate significant difference at *p* < 0.05. PDI: polymer dispersity index; FJ: fresh juice; TS0: thawed Huyou juice without ultrasonic treatment and storage; US0: thawed Huyou juice after ultrasonic treatment with ultrasonic power of 360 W for 30 min and without storage.

**Table 2:** The effect of ultrasonic treatment and storage on the color, total soluble solids (TSS) and titratable acidity (TA) of thawed Huyou juice.

Treatment	L*	a*	b*	TSS/° Brix	TA/%
TS0	21.33 ± 5.84 ^a^	−1.31 ± 0.48 ^b^	20.45 ± 7.55 ^a^	11.7 ± 0.2 ^b^	0.76 ± 0.03 ^b^
TS7	21.08 ± 3.29 ^a^	−1.96 ± 0.48 ^b^	18.58 ± 4.91 ^a^	11.5 ± 0.2 ^a,b^	0.74 ± 0.02 ^a,b^
US0	23.85 ± 2.80 ^a^	−4.19 ± 0.13 ^a^	17.74 ± 4.37 ^a^	11.2 ± 0.2 ^a^	0.72 ± 0.01 ^a,b^
US7	25.01 ± 1.71 ^a^	−4.31 ± 0.06 ^a^	18.65 ± 2.81 ^a^	11.0 ± 0.2 ^a^	0.69 ± 0.02 ^a^

Mean ± SD indicated three replicates. Superscripts of different letters indicate significant difference at *p* < 0.05. TS0: thawed Huyou juice without ultrasonic treatment and storage; TS7: thawed Huyou juice without ultrasonic treatment after 7 days of storage; US0: thawed Huyou juice after ultrasonic treatment with ultrasonic power of 360 W for 30 min and without storage; US7: thawed Huyou juice after ultrasonic treatment with ultrasonic power of 360 W for 30 min and with 7 days of storage at 4 °C.

**Table 3:** The effect of ultrasonic treatment and storage on of bioactive compounds of thawed Huyou juice.

Treatment	DPPH Radical Scavenging (AAE mg/L)	FRAP (TE g/L)	Total Phenolics (GAE mg/L)
TS0	419.11 ± 16.79 ^a^	77.84 ± 6.99 ^a^	231.75 ± 3.22 ^b^
TS7	407.27 ± 21.46 ^a^	88.83 ± 15.56 ^a^	231.64 ± 10.51 ^b^
US0	416.53 ± 9.37 ^a^	93.94 ± 7.30 ^a^	224.23 ± 3.25 ^a^
US7	407.60 ± 10.00 ^a^	85.24 ± 9.96 ^a^	226.11 ± 7.30 ^a,b^

Mean ± SD indicated three replicates. Superscripts of different letters indicated significant difference at *p* < 0.05. TS0: thawed Huyou juice without ultrasonic treatment and storage; TS7: thawed Huyou juice without ultrasonic treatment after 7 days of storage; US0: thawed Huyou juice after ultrasonic treatment with ultrasonic power of 360 W for 30 min and without storage; US7: thawed Huyou juice after ultrasonic treatment with ultrasonic power of 360 W for 30 min and with 7 days of storage; TE: trolox equivalent; AAE: ascorbic acid equivalent; GAE: gallic acid equivalent.

**Table 4:** The effect of ultrasonic treatment and storage on flavanone composition of thawed Huyou juice.

Treatment	Total Flavonoids (RE mg/L)	Flavanone Composition (mg/L)				
		Eriocitrin	Neoeriocitrin	Narirutin	Naringin	Neohesperidin
TS0	255.56 ± 9.33 ^a^	74.52 ± 1.13 ^a^	42.61 ± 0.77 ^a^	336.51 ± 3.49 ^a^	332.39 ± 3.91 ^a^	96.76 ± 1.76 ^a^
TS7	258.24 ± 8.92 ^a^	68.52 ± 3.91 ^a^	40.93 ± 2.26 ^a^	329.81 ± 11.95 ^a^	330.36 ± 25.61 ^a^	95.80 ± 4.30 ^a^
US0	261.68 ± 12.29 ^a^	68.92 ± 2.33 ^a^	39.54 ± 1.66 ^a^	320.41 ± 7.81 ^a^	310.82 ± 17.31 ^a^	90.16 ± 2.57 ^a^
US7	268.10 ± 9.85 ^a^	72.33 ± 4.26 ^a^	41.70 ± 0.95 ^a^	333.02 ± 11.45 ^a^	318.30 ± 4.58 ^a^	95.15 ± 4.25 ^a^

Mean ± SD indicated three replicates. Superscripts of different letters indicated significant difference at *p* < 0.05. TS0: thawed Huyou juice without ultrasonic treatment and storage; TS7: thawed Huyou juice without ultrasonic treatment after 7 days of storage; US0: thawed Huyou juice after ultrasonic treatment with ultrasonic power of 360 W for 30 min and without storage; US7: thawed Huyou juice after ultrasonic treatment with ultrasonic power of 360 W for 30 min and with 7 days of storage; RE: rutin equivalent.

## Data Availability

The data presented in this study are available on request from the corresponding author. The data are not publicly available due to privacy reasons.

## Supplementary Materials

The following are available online at https://www.mdpi.com/article/10.3390/foods10081695/s1, Figure S1: HPLC chromatograms of five flavonoid standards and flavonoids in samples, Figure S2: Appearance of fresh Huyou juice during storage.
