# Supplementary material for: Ultrasonication of Thawed Huyou Juice: Effects on Cloud Stability, Physicochemical Properties and Bioactive Compounds
Source: Foods. 2021 Jul 22;10(8):1695. doi: 10.3390/foods10081695 (PMC8391196; doi:10.3390/foods10081695)
Supplement: Supplementary file 1 [file foods-10-01695-s001.zip › foods-1280982-supplementary.pdf]

Supplemental materials

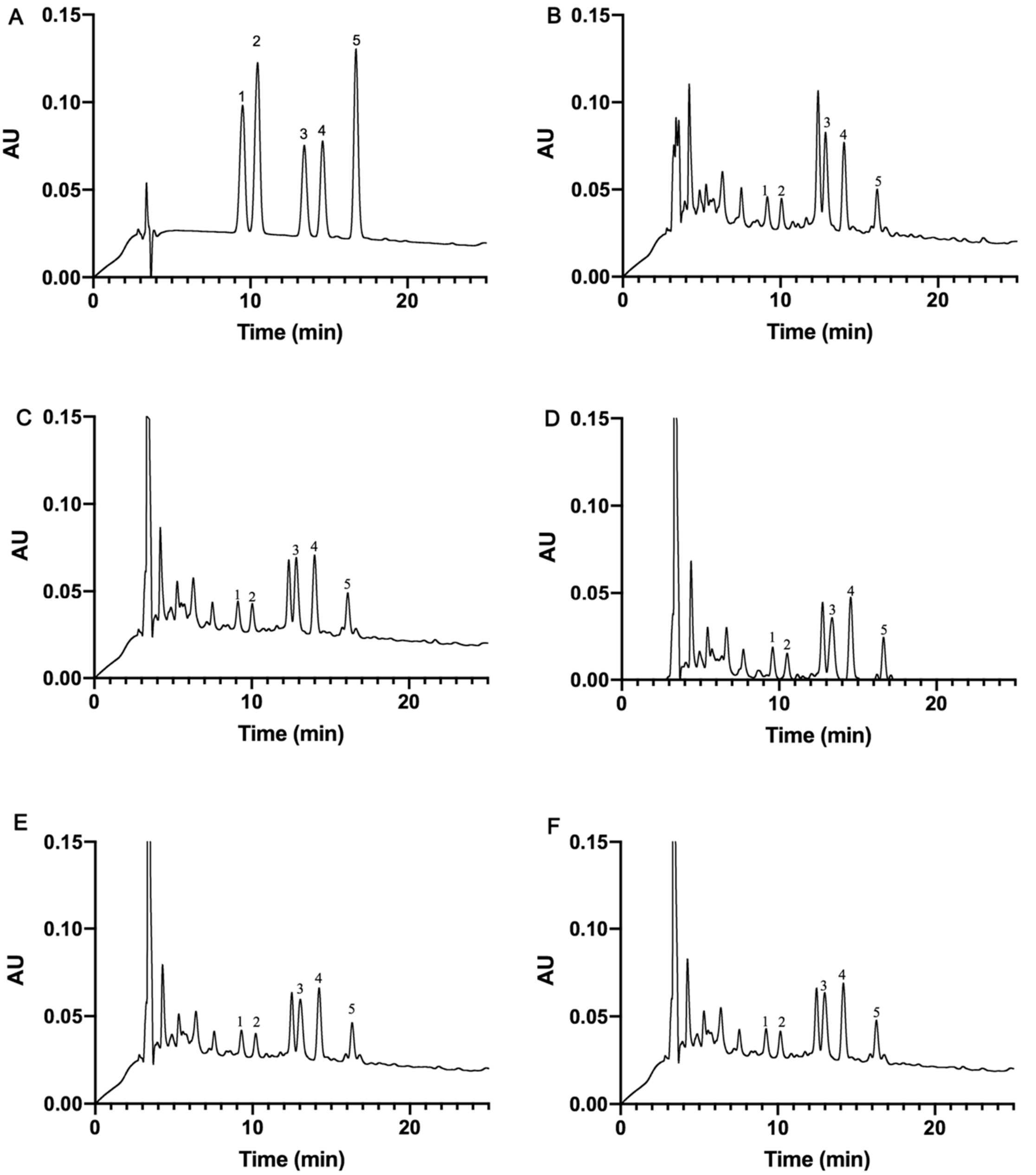

Figure S1. HPLC chromatograms of five flavonoid standards and flavonoids in samples.

(A) Flavonoid standard solution; (B) Fresh Huyou juice; (C) Thawed Huyou juice without ultrasonication stored for 0 days; (D) Thawed Huyou juice without ultrasonication stored for 7 days; (E) Thawed Huyou juice which was treated with ultrasonic power of 360 W for 30 min and then stored for 0 days; (F) Thawed Huyou juice which was treated with ultrasonic power of 360 W for 30 min and then stored for 7 days.

Peak numbers: 1-Eriocitrin; 2-Neoeriocitrin; 3-Narirutin; 4)-Naringin; 5-Neohesperidin.

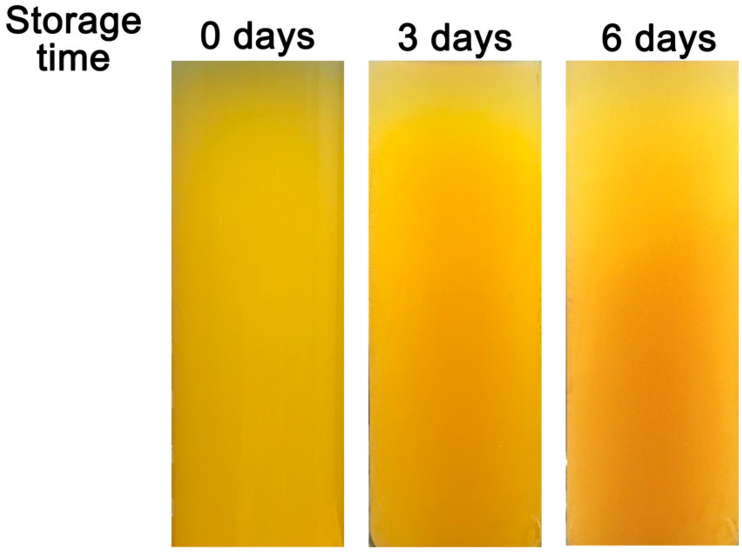

Figure S2. Appearance of fresh Huyou juice during storage.
